# A Novel Human Systemic Lupus Erythematosus Model in Humanised Mice

**DOI:** 10.1038/s41598-017-16999-7

**Published:** 2017-11-30

**Authors:** Merry Gunawan, Zhisheng Her, Min Liu, Sue Yee Tan, Xue Ying Chan, Wilson Wei Sheng Tan, Shubasree Dharmaraaja, Yong Fan, Chee Bing Ong, Eva Loh, Kenneth Tou En Chang, Thiam Chye Tan, Jerry Kok Yen Chan, Qingfeng Chen

**Affiliations:** 1 Humanized Mouse Unit, Institute of Molecular and Cell Biology, Agency for Science, Technology and Research (A*STAR), Singapore, Singapore; 2 Key Laboratory for Major Obstetric Diseases of Guangdong Province, The Third Affiliated Hospital of Guangzhou Medical University, Guangzhou, China; 3 Advanced Molecular Pathology Laboratory, Institute of Molecular and Cell Biology, Agency for Science, Technology and Research (A*STAR), Singapore, Singapore; 4 Department of Pathology and Laboratory Medicine, KK Women’s and Children’s Hospital, Singapore, Singapore; 5 Department of Obstetrics & Gynaecology, KK Women’s and Children’s Hospital, Singapore, Singapore; 6 Department of Reproductive Medicine, KK Women’s and Children’s Hospital, Singapore, Singapore; 7 Experimental Fetal Medicine Group, Yong Loo Lin School of Medicine, National University of Singapore, Singapore, Singapore; 8 Department of Microbiology and Immunology, Yong Loo Lin School of Medicine, National University of Singapore, Singapore, Singapore

## Abstract

Mouse models have contributed to the bulk of knowledge on Systemic Lupus Erythematosus (SLE). Nevertheless, substantial differences exist between human and mouse immune system. We aimed to establish and characterise a SLE model mediated by human immune system. Injection of pristane into immunodeficient mice reconstituted with human immune system (humanised mice) recapitulated key SLE features, including: production of human anti-nuclear autoantibodies, lupus nephritis, and pulmonary serositis. There was a reduction in the number of human lymphocytes in peripheral blood, resembling lymphopenia in SLE patients. Concurrently, B cells and T cells were systemically hyperactivated, with a relative expansion of CD27^+^ and CD27^−^IgD^−^ memory B cells, increased number of plasmablasts/plasma cells, and accumulation of effector memory T cells. There was also an increased production of human pro-inflammatory cytokines, including: IFN-γ, IL-8, IL-18, MCP-1, and IL-6, suggesting their role in SLE pathogenesis. Increased expression of type I IFN signature genes was also found in human hepatocytes. Altogether, we showed an SLE model that was mediated by human immune system, and which recapitulated key clinical and immunological SLE features. The advancements of humanised mice SLE model would provide an *in vivo* platform to facilitate translational studies and pre-clinical evaluations of human-specific mechanisms and immunotherapies.

## Introduction

Systemic Lupus Erythematosus (SLE) is a chronic, relapsing autoimmune disorder where the immune system targets multiple self-nuclear antigens, leading to chronic organ damage and mortality^1^. The systemic nature of SLE is manifested in a highly heterogenous manner, such that the Systemic Lupus Collaborating Clinics (SLICC) established 17 criteria for SLE classification, including both clinical and immunological criterion^2^. Clinical manifestations of SLE involve multiple organs, ranging from skin rash, neurologic dysfunction, joint synovitis, serositis and renal inflammation, known as lupus nephritis. There is no cure for SLE, and current treatments for SLE mostly relied on empirical use of NSAIDs and immunosuppressants to manage symptoms associated with SLE. Only one FDA-approved treatment targeting B cell anomalies in patients with active SLE has emerged in the past 55 years^3^. As such, the need for SLE treatment to reduce mortality and morbidity remains critical.

The exact etiology of SLE remains unknown, and the disease is thought to derive from multiple factors, including genetic predispositions, environmental and hormonal factors. Study of human SLE face many challenges owing to the complex nature of SLE, and the lack of definitive diagnostic and prognostic biomarkers of disease activity^4,5^. Moreover, human studies are generally restricted by ethical limitations to *in vitro* or *ex vivo* assays. Animal models, particularly murine, have contributed to the bulk of knowledge regarding the etiopathogenesis of SLE^6^. Spontaneous models using inbred strains, such as the NZB/W F1 mice^7^, MRL/lpr mice^8^, and BXSB/Yaa mice models^9^, possess genetic backgrounds that confers SLE susceptibility, and develop spontaneous nephritis and autoantibodies production. These spontaneous models have been particularly useful in studying the complex genetic contribution in SLE. In addition to the spontaneous models, SLE can be induced in different mice strains through a number of ways, including induced Graft versus host disease^10^, as well as injection of a synthetic mineral oil known as pristane (Tetramethylpentadecane, TMPD)^11^. Single pristane injection into various mouse strains could induce most histopathological features of SLE, and it is one of the few animal SLE models to exhibit the type I interferon (IFN) signature genes (ISG) expression, as is observed in SLE patients^12^. Despite the non-spontaneous nature, induced SLE model is particularly beneficial in determining the contribution of single gene/factor in SLE pathogenesis, which would require significant time and resources to backcross onto the spontaneous SLE strains.

While various mouse models have provided fundamental insights on SLE pathogenesis, they have not fully recapitulated the whole spectrum and complexity of human SLE. Importantly, substantial differences exist between mouse and human immune system^13,14^. Findings in mouse models may not be directly translatable to human, and have to be taken with caution, particularly in the development and evaluation of therapeutic protocols. The use of humanised mice (from hereon referred to as hu-mice), where human immune system is stably reconstituted into immunodeficient mice, has allowed *in vivo* studies of human immunology, particularly for human-specific infectious diseases and cancer^15^. However, the use of hu-mice for the study of human autoimmune diseases remained largely unsuccessful^16^. Several attempts to study the pathogenesis of human SLE in immunodeficient mice have been described, through engraftment of peripheral blood mononuclear cells (PBMCs) from SLE patients into immunodeficient mice^17–19^. Nevertheless, these previous models were limited by low efficiency of human cells engraftment, requirement of large numbers of PBMCs from SLE patients who are often lymphopenic, or the lack of human anti-nuclear autoantibodies production, which is widely considered as one of the hallmark symptoms of SLE. Here we described a model of human immune system-mediated SLE induced by pristane injection in hu-mice. Most of the clinical and immunological features of human SLE, including production of human anti-nuclear autoantibodies, pulmonary serositis, lupus nephritis, proteinuria, lymphopenia, lymphocytes hyperactivation and aberrant cytokine production could be recapitulated. A model of SLE induced in human immune system would allow numerous *in vivo* studies of human-specific SLE pathogenesis in manners that is not possible in human. Importantly, it would facilitate translational studies for the development and evaluation of targeted immunotherapy for human SLE.

## Results

### Early mortality in pristane-injected hu-mice

Engraftment of human CD34^+^ haematopoietic stem cells (HSCs) into sublethally irradiated NOD/LtSz-Prkdc^scid^Il2rg^tm1whl^/J (NSG) pups consistently achieved good human immune system reconstitution, with an average of 42.12% (±1.296 S.E.M) reconstitution level in the blood, and higher in the tissues such as spleen (82.83% ± 2.77 S.E.M), mesenteric lymph nodes (97.39% ± 0.42 S.E.M), and liver (89.03% ± 1.98 S.E.M) (Supplementary Fig. S1A).

**Figure 1:**
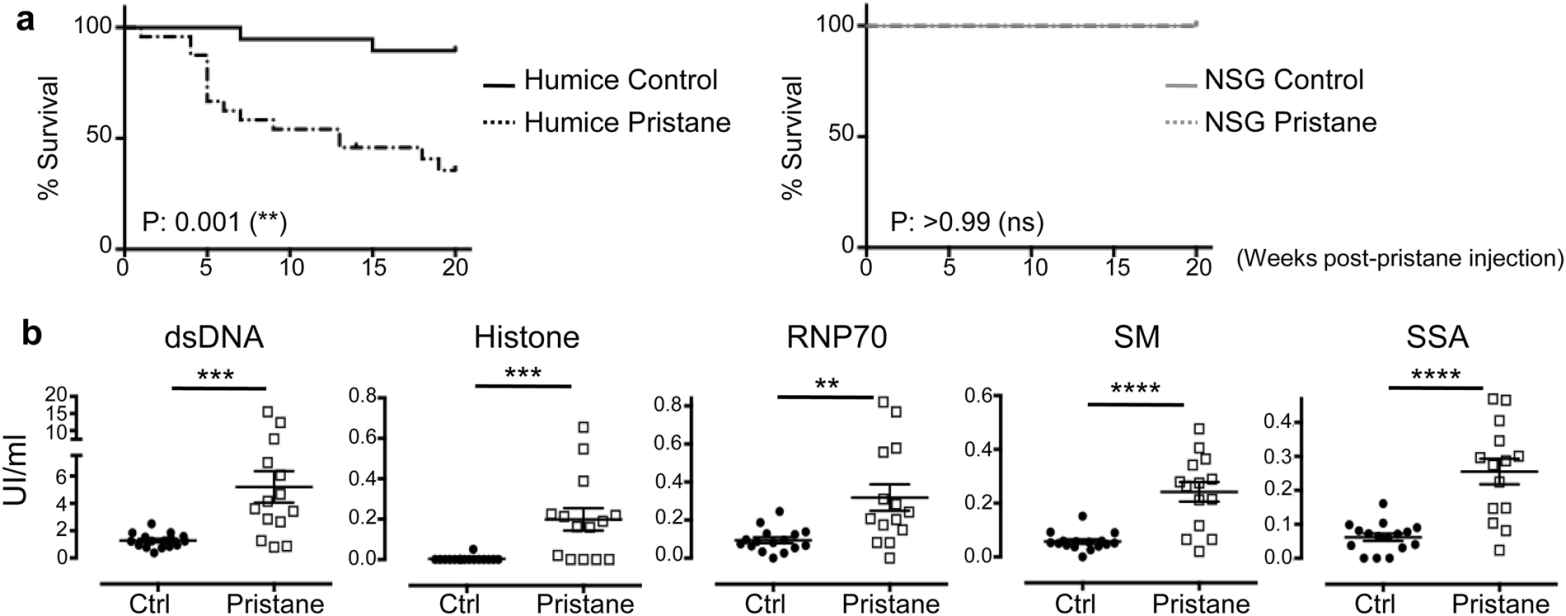
Increased mortality and production of human anti-nuclear autoantibodies in pristane-injected hu-mice. (**a**) Survival curve of hu-mice (left) and NSG (right), with or without pristane injection, as indicated, was monitored for 20 weeks after treatment. Figure shown is from three independent experiments (control n = 19; pristane n = 24; NSG control n = 9; NSG pristane n = 9). (**b**) Level of human anti-nuclear autoantibodies in blood plasma were measured by ELISA at 8 weeks post-pristane injection in hu-mice. Control group represents data from littermate hu-mice without pristane injection. Data shown is from four independent experiments (control n = 16; pristane n = 14). **P < 0.01; ***P < 0.001.

NSG mice completely lack T, B and NK cells. However, residual mouse myeloid cells remain, albeit with impaired function^20^. To examine if these residual mouse cells could contribute to SLE pathogenesis in hu-mice, pristane was injected to both NSG and hu-mice. Pristane-injected NSG mice appeared healthy, and there was no mortality throughout the observation period of 20 weeks post injection. On the other hand, pristane injection into hu-mice induced significantly earlier mortality with a median survival at 13 weeks post injection (Fig. 1A). The absolute cell number counts and reconstitution levels of human CD45^+^ leukocytes, CD3^+^ T cells, CD19^+^ B cells, CD4^+^ T cells and CD8^+^ T cells in control and pristane treated hu-mice were shown in Supplementary Fig. S1B. This showed that the human immune system is necessary and sufficient for the induction of SLE pathogenesis, leading to increased mortality in hu-mice; and, on the other hand, the residual mouse myeloid cells did not contribute significantly to the pathogenesis.

### Induction of autoantibodies production

SLE is characterised by the presence of a spectrum of autoantibodies against nuclear antigens. Pristane-induced SLE model produced the widest range of autoantibodies compared to other spontaneous SLE mouse models^12^. Correspondingly, pristane injection into hu-mice increased the total level of human IgG and IgM (Supplementary Fig. S2A), and induced production of a wide range of human anti-nuclear autoantibodies, including anti-dsDNA, anti-histone, anti-RNP70, anti-SM and anti-SSA IgGs (Fig. 1B). Increased production of human anti-dsDNA IgG could be detected significantly as early as four weeks post injection, and continued to increase to week 8 (Supplementary Fig. S2B). There was no significant difference in the level of anti-dsDNA IgG in female and male pristane-injected hu-mice (Supplementary Fig. S2C).

### Lupus Nephritis and Pleuritis

**Figure 2:**
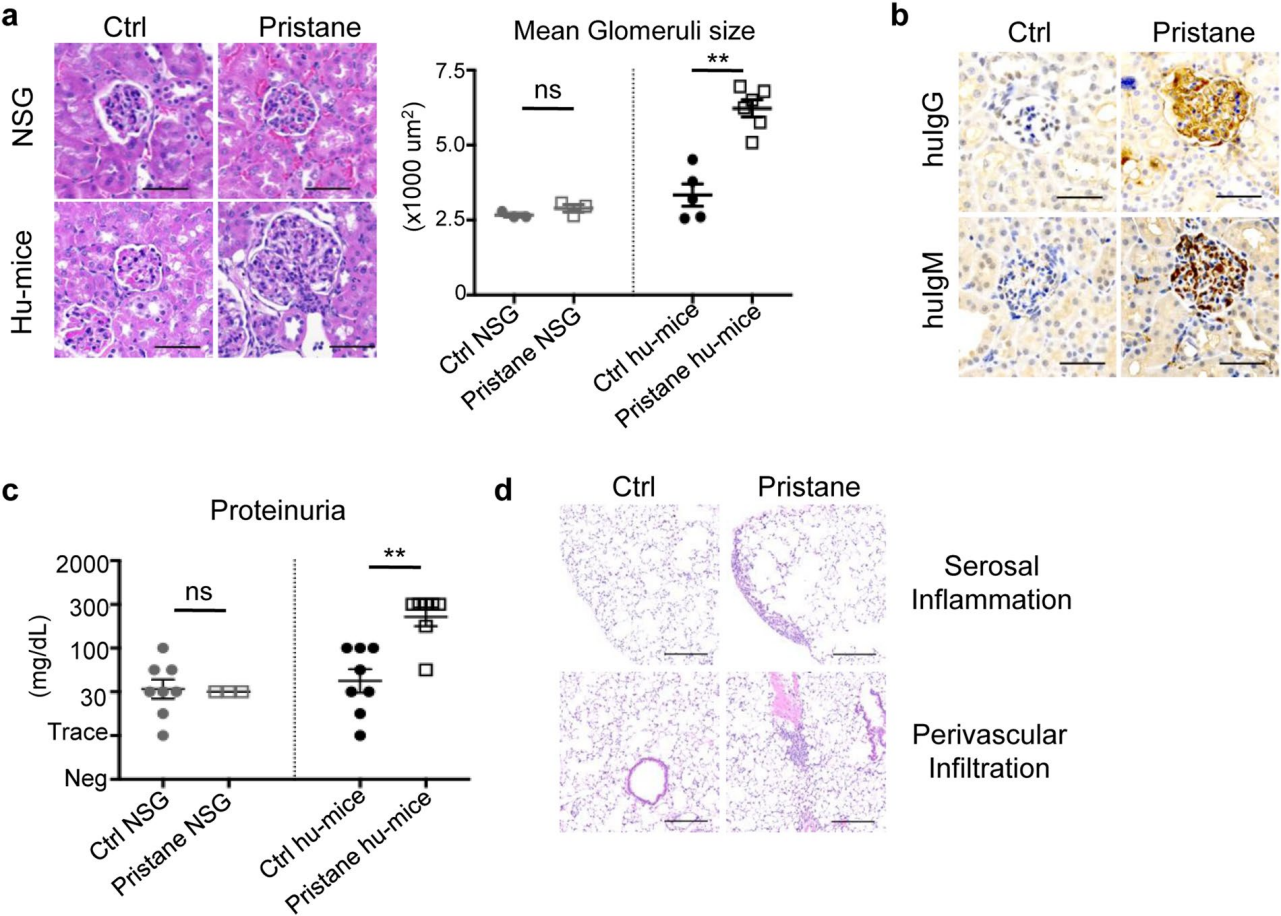
Lupus nephritis and pulmonary inflammation. (**a**) (Left) Kidney sections from hu-mice and NSG with or without pristane injection were H&E stained and pathologically evaluated. Scale bar represents 50 µm and images are representative from two independent experiments (control NSG n = 3; pristane NSG n = 3; control hu-mice n = 8; pristane hu-mice n = 12). (Right) Mean glomeruli area was measured from 50 random glomeruli from kidneys of each experimental animal (control NSG n = 3; pristane NSG n = 3; control hu-mice n = 5; pristane hu-mice n = 6). (**b**) Immunohistochemistry of human IgG and IgM on kidney sections from control and pristane-injected hu-mice Scale bar represents 50 µm and images are representative from two independent experiments (control n = 8; pristane n = 12). (**c**) Protein content in the urine of control and pristane-injected NSG, control and pristane-injected hu-mice at 10 weeks post-pristane injection was measured using Uristix reagent strips. Figure is from two independent experiments (control NSG n = 8; pristane NSG n = 3; control hu-mice n = 8; pristane hu-mice n = 7). (**d**) Lung sections were H&E stained and pathologically evaluated. Scale bar represents 200 µm and images are representative from two independent experiments (control n = 6; pristane n = 5). **P < 0.01.

Lupus nephritis, or kidney inflammatory condition associated with SLE, affects the majority of SLE patients, and kidney failure is one of the leading causes of mortality in individuals with active SLE^21^. Lupus nephritis is characterised by the presence of immune deposits in the glomeruli, which induced leukocyte infiltration and severe damage, leading to protein leakage into the urine, or proteinuria^22^. Histological evaluation of kidneys from pristane-injected hu-mice revealed kidney inflammation and glomerular changes that are consistent with proliferative glomerulonephritis. Specifically, there was focal to diffuse global glomerular enlargement by mesangial/endocapillary proliferation and increased glomerular cellularity, which was not evident in the kidneys of pristane-injected NSG mice (Fig. 2A). Human leukocyte infiltration was further verified by immunohistochemistry staining of human CD45^+^ cells in the glomeruli of pristane-injected hu-mice (Supplementary Fig. S2D). Consistent with immune complex deposition observed in SLE patients, human IgG and IgM deposition was found in the glomeruli of pristane-injected hu-mice (Fig. 2B). Expectedly, this was accompanied by increased proteinuria in pristane-injected humice, which was not observed in pristane-injected NSG (Fig. 2C). The presence of kidney damage and proteinuria in pristane-injected hu-mice, but not pristane-injected NSG mice, again implicates the key role of human immune system, and the minimal contribution of residual mouse cells, in the induction of SLE pathogenesis upon pristane injection.

In addition to nephritis, pulmonary manifestation affects up to 50% of individuals with SLE. The most common pulmonary manifestation associated with SLE is lung serositis (inflammation in the lining of the lung, known as pleuritis) and interstitial lung inflammation^23^. Pathological evaluation of lung from pristane-injected hu-mice revealed increased multifocal serosal and subpleural inflammation with fibrosis, as well as perivascular interstitial and intra-alveolar mononuclear cell infiltrate (Fig. 2D). Altogether, the presence of lupus nephritis, pulmonary serositis, and autoantibodies production showed that pristane injection could induce SLE pathogenesis that is mediated by the human immune system in hu-mice.

### Lymphopenia in pristane-injected hu-mice

**Figure 3:**
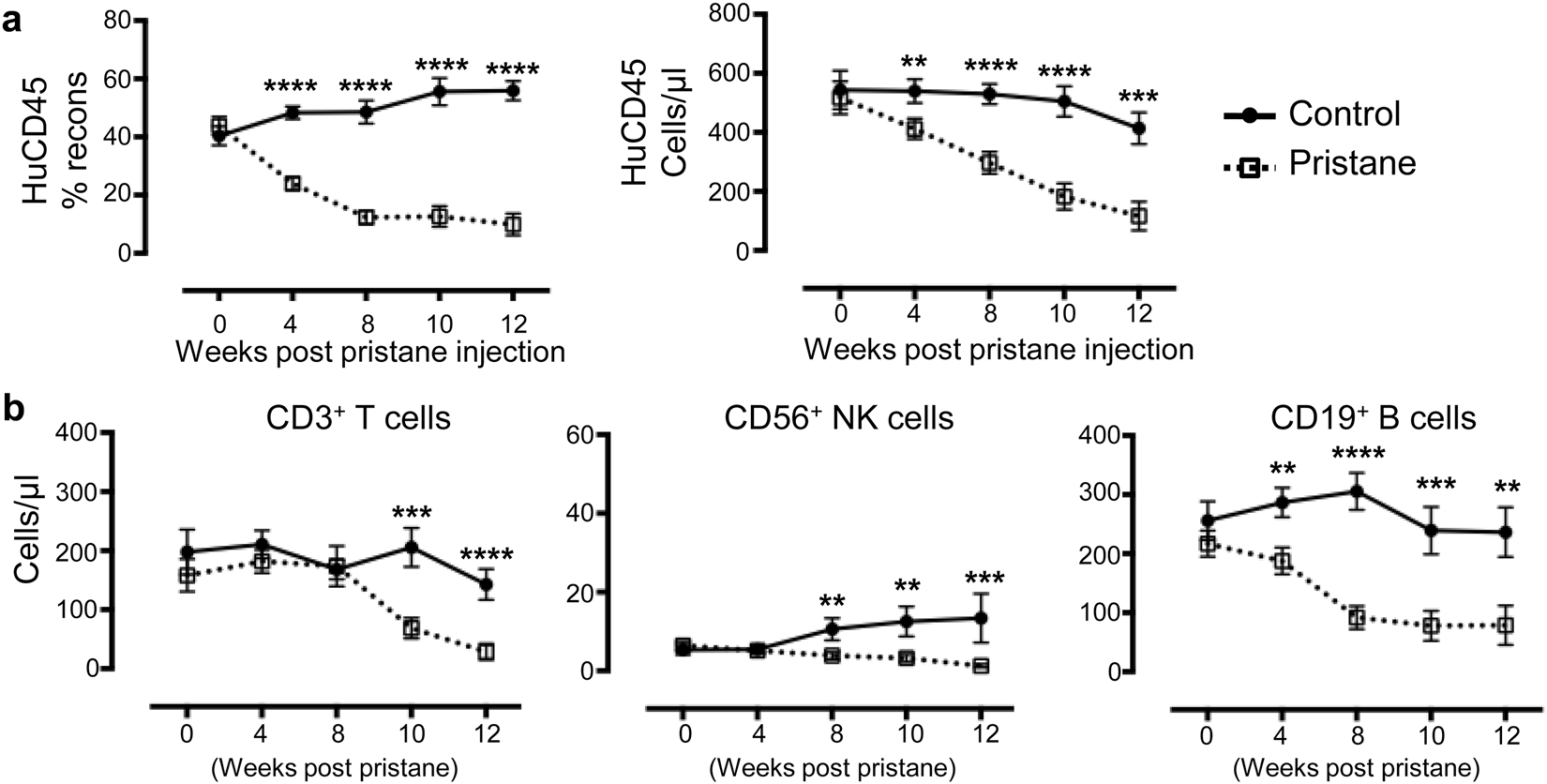
Lymphopenia in pristane-injected hu-mice. (**a**) Reconstitution level (left) and absolute number (right) of human immune cells in the peripheral blood of control and pristane-injected hu-mice were measured by flow cytometry at the indicated time points. (**b**) Absolute number of human T cells, B cells and NK cells in the peripheral blood was measured based on surface expression of human CD3, CD19 and CD56, respectively. Absolute cell numbers/μl blood was calculated using counting beads (Materials and Methods). Data shown was from four independent experiments (control n = 20; pristane n = 26). **P < 0.01; ***P < 0.001; ****P < 0.0001.

Lymphopenia is a common feature and one of the diagnostic criteria in SLE^24^. It, however, is not often recapitulated in most SLE animal models; instead, many well-studied SLE mouse models displayed massive lymphoproliferation^6^. Pristane injection into hu-mice induced a dramatic drop in the reconstitution percentage and absolute number of human immune cells in the blood over a period of 12 weeks (Fig. 3A; Supplementary Fig. S3A). Consistent with lymphopenia observed in SLE patients^25^, there was a significant reduction in the absolute number of human T cells, B cells and NK cells. B cell numbers were significantly reduced as early as four weeks post pristane injection, while the number of T cells and NK cells were reduced at a later stage at week 8 and 10, respectively (Fig. 3B). Notably, pristane injection to hu-mice induced an accumulation of both human and mouse immune cells in the peritoneal lavage (Supplementary Fig. S3B), and a mild spleen enlargement with a significant increase in the number of mouse, but not human immune cells (Supplementary Fig. S3C,D).

### Lymphocyte hyperactivation in hu-mice

**Figure 4:**
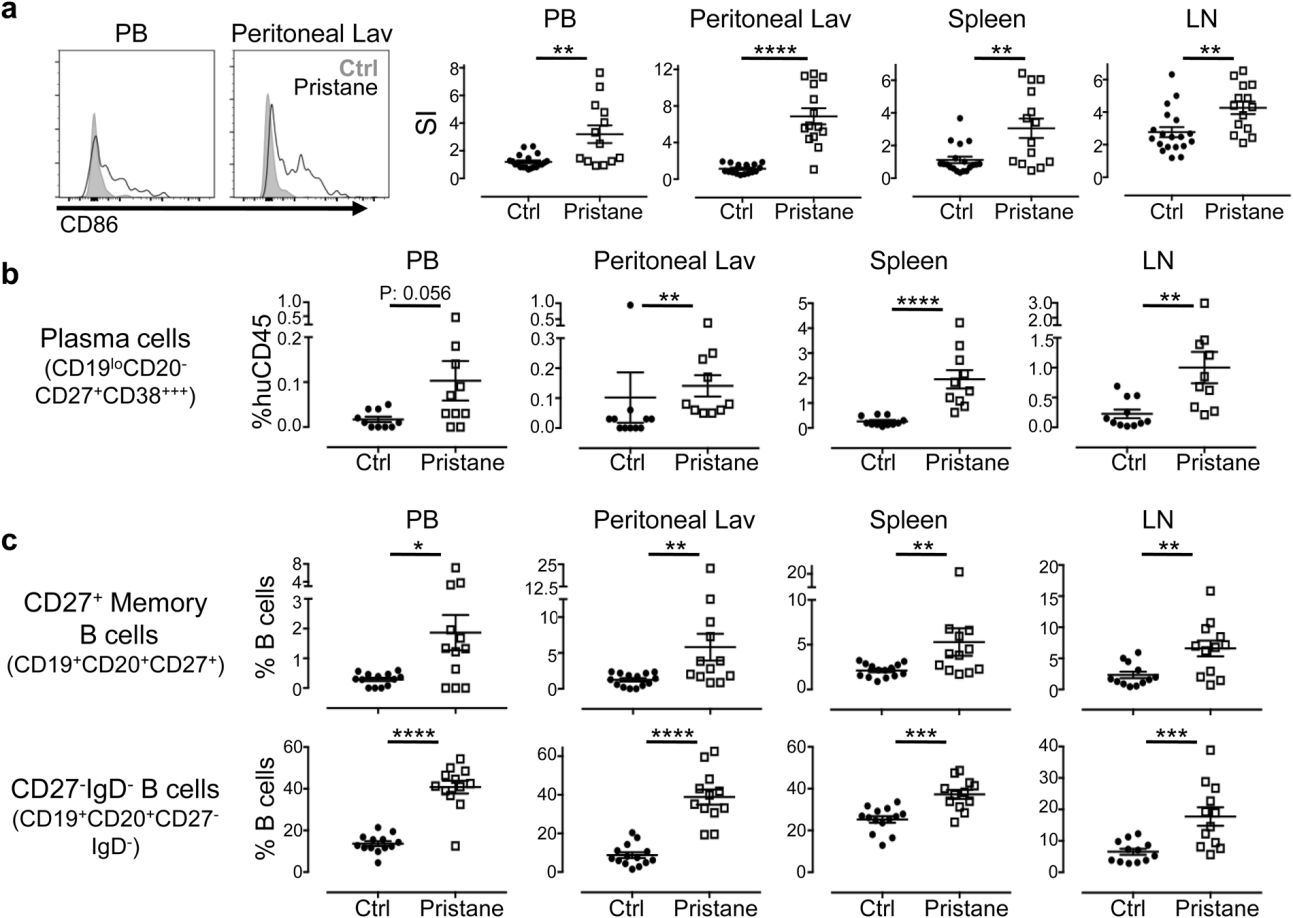
B cell hyperactivation and anomalies. (**a**) Human B cell in the peripheral blood (PB), peritoneal lavage (lav), spleen and lymph node (LN) of control and pristane-injected hu-mice were analysed by FACS at 8 weeks after pristane injection. B cells activation was measured by expression of B cell activation marker, CD86. Histogram (left) showed representative staining of CD86 on CD19^+^CD20^+^ B cells in PB and peritoneal lavage of control and pristane-injected hu-mice. Relative expression level was quantified as Staining Index (right) Figure shown is from four independent experiments (control n = 18; pristane n = 14). (**b**) Human plasma cells/plasmablasts were identified by their surface markers CD19^lo^CD20^−^CD27^+^CD38^hi^ and were quantified as percentage of human CD45. Figure is from three independent experiments (control n = 11; pristane n = 10). (**c**) CD19^+^CD20^+^ B cell subsets were identified by their surface markers CD27 and IgD, as indicated, and were quantified as percentage of CD19^+^CD20^+^ B cells. Figure shown is from three independent experiments (control n = 14; pristane n = 12). *P < 0.05; **P < 0.01; ***P < 0.001; ****P < 0.0001.

As antibody producing cells, B cells have been the focus of many studies and targeted immunotherapies for SLE, not only for their role in producing autoantibodies, but also in antigen presentation and cytokine expression^26^. Various B cell anomalies have been reported in SLE patients, including lymphopenia, hyperactivation, relative expansion of memory B cells, as well as increased frequency of antibody-producing plasma cells^26^. To determine B cell abnormality in pristane-injected hu-mice, B cell subsets and activation markers in peripheral blood as well lymphoid organs were analysed by flow cytometry. Indeed, pristane injection induced systemic B cell hyperactivation, as indicated by the increased expression of B cell activation marker CD86, in peripheral blood, spleen, mesenteric lymph nodes and peritoneal lavage (Fig. 4A). There was increased frequency (Fig. 4B and Supplementary Fig. S4A) and number (Supplementary Fig. S4B) of CD19^+^CD20^−^CD27^hi^CD38^hi^ plasmablasts/plasma cells in the peripheral blood and spleen of pristane-injected hu-mice. The CD27^−^IgD^−^ B cell population has been reported to consist of a distinct memory B cells population, and is highly expanded in SLE patients^27^. Parallel to the observation in SLE patients, there was a relative expansion in the frequency of CD27^+^ memory B cells and CD27^−^IgD^−^ B cell population (Fig. 4C); and a reduction in the CD27^−^IgD^+^ naïve/transitional B cells compartment (Supplementary Fig. S4C).

**Figure 5:**
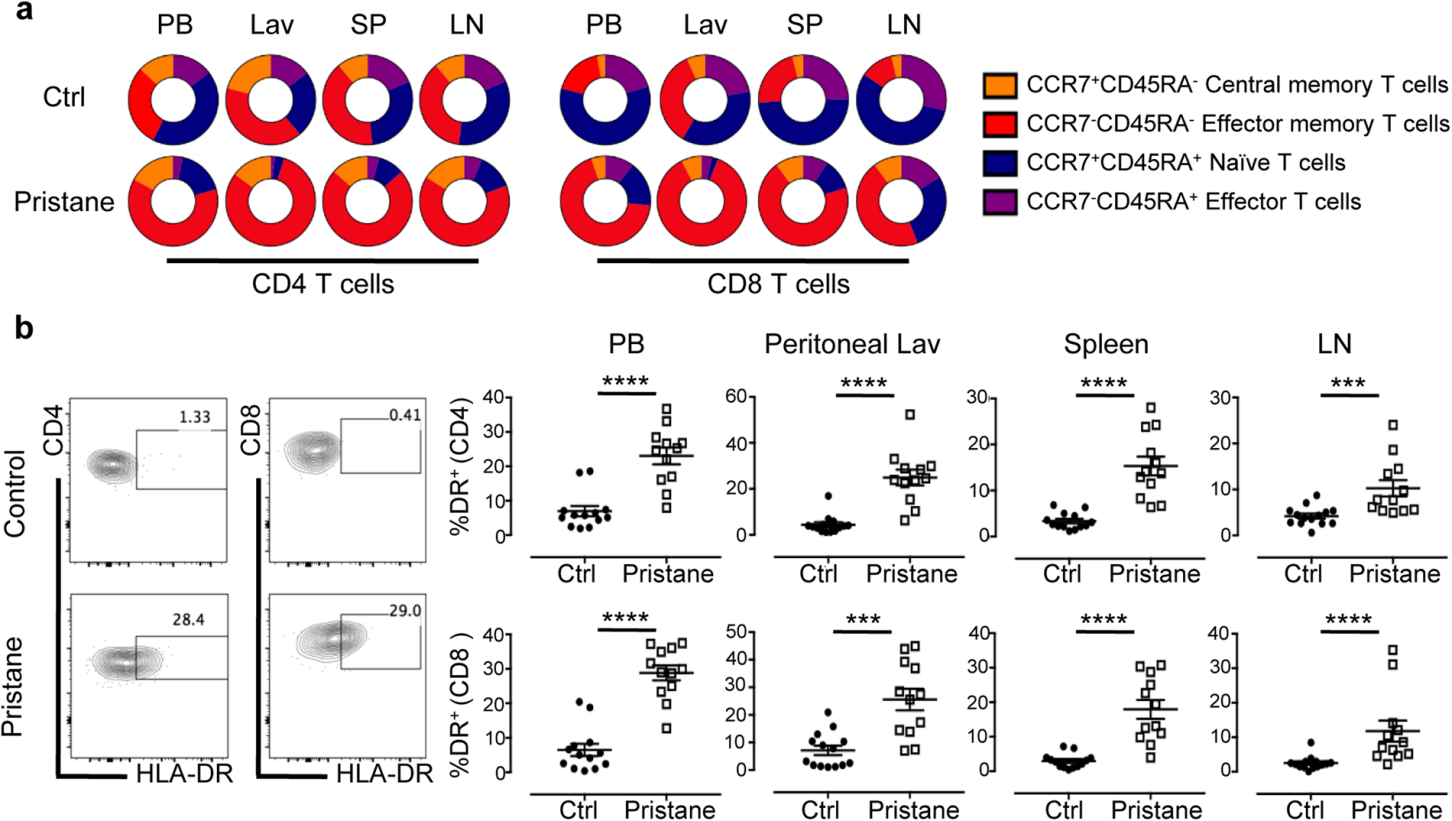
T cell hyperactivation and accumulation of memory cells. (**a**) Human T cells in the peripheral blood (PB), peritoneal lavage (Lav), spleen (SP) and lymph node (LN) of control and pristane-injected hu-mice were analysed by FACS at 8 weeks after pristane injection. Proportion of human CD4 and CD8 naïve, effector, and memory T cells were quantified according to the surface expression of CCR7 and CD45RA, as indicated. Pie chart shown is the mean value from four independent experiments (control n = 18; pristane n = 14). Statistical analysis is attached in Supplementary Table S1. (**b**) (Left) Flow plot showed representative staining of HLA-DR in CD4 and CD8 T cells in the PB. (Right) Percentage of HLA-DR expressing cells were quantified from the total CD4 or CD8 T cells population, as indicated. Figure shown is from three independent experiments (control n = 14; pristane n = 12). ***P < 0.001; ****P < 0.0001.

In addition to its role in supporting antibodies production, T cells majorly contribute to the inflammatory response in SLE through inflammatory cytokine production and tissue infiltrations that often lead to organ damage^28^. Various signaling abnormalities in T cells lead to chronic T cell activation, reduced peripheral tolerance, and accumulation of autoreactive memory T cells^29^. Similar to patients with active SLE, pristane injection in hu-mice induced activation of both CD4^+^ and CD8^+^ T cells^30,31^. There was a marked reduction of both CD4^+^ and CD8^+^ T cells with naïve phenotype (CCR7^+^CD45RA^+^), and increased proportion of T cells with an effector memory phenotype (CCD7^−^CD45RA^−^) in the peripheral blood, spleen, mesenteric lymph nodes and peritoneal lavage (Fig. 5A), indicating a systemic inflammation condition. Accordingly, T cell activation was also evident in the increased proportion of both CD4^+^ and CD8^+^ T cells expressing HLA-DR (Fig. 5B), a human-specific lymphocyte activation marker^32^. Hence, we conclude that both T cell and B cell activation play a significant role in the pathogenesis of induced SLE in hu-mice. Moreover, SLE induction in hu-mice exhibited lymphocyte anomalies with high resemblance to the anomalies observed in SLE patients.

### Production of human pro-inflammatory cytokines

**Figure 6:**
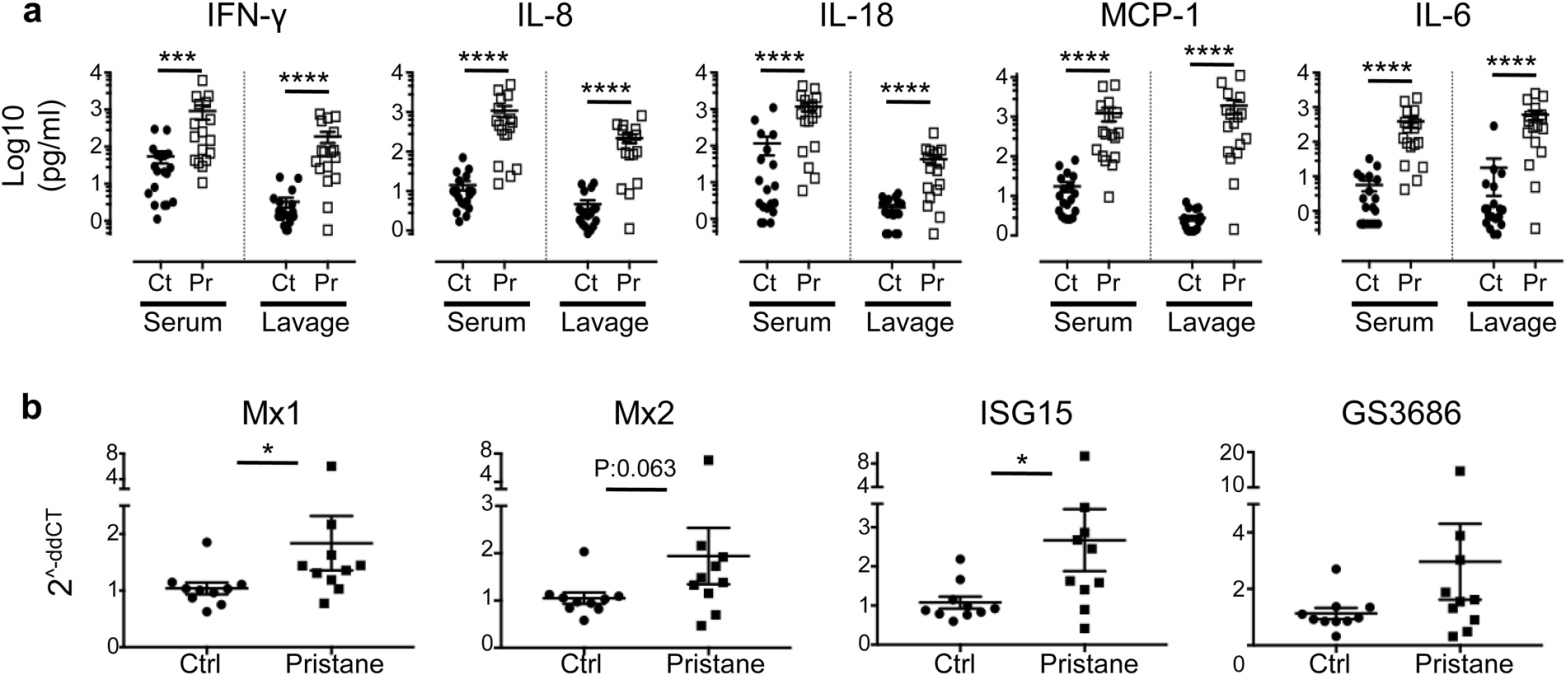
Aberrant production of human pro-inflammatory cytokines. (**a**) Levels of human proinflammatory cytokines, as indicated, in the blood serum and peritoneal lavage fluid (lavage) of control and pristane-injected hu-mice were measured by a multiplex bead-based immunoassay at 8 weeks after pristane injection. Figure shown is from five independent experiments (control n = 19; pristane n = 17).Ct: control; Pr: pristane. (**b**) Gene expression level of type I IFN target genes in human hepatocytes in control and pristane-injected humice at four weeks after pristane injection. Data is representative of three independent experiments (control n = 10; pristane n = 10). *P < 0.05; ***P < 0.001; ****P < 0.0001

Both T cells and B cells contribute to SLE pathogenesis through aberrant cytokines production, which modulates the innate and adaptive immune responses^33^. To measure the involvement of human cytokines in pristane-induced SLE in hu-mice, human pro-inflammatory cytokine levels were measured in blood plasma as well as peritoneal lavage fluid. There was a significant increase in the level of inflammatory cytokines such as IFN-γ, IL-18, IL-8, MCP-1 and IL-6 (Fig. 6A), which indicates their role in the SLE pathogenesis.

Furthermore, it has been shown in our previous publications that with the transplantation of human fetal liver CD34^+^ cells, humanized mice develop a ~5% chimerism of human hepatocytes in the liver^34,35^. Consistent with the expression of type I IFN signature genes (ISG) expression in mouse pristane model, we observed increased expression of human ISGs (Mx1, Mx2, and ISG15) in the human hepatocytes of pristane-injected hu-mice (Fig. 6B). Taken together, systemic lymphocytes hyperactivation coupled with aberrant production of pro-inflammatory cytokines and increased expression of ISGs, suggests that pristane injection into hu-mice induces a chronic systemic inflammation similar as was seen in SLE.

## Discussion

Hu-mice containing stable engraftment of human immune system in immunodeficient mouse have proven to be instrumental in the study of human immunology, particularly for human-specific infectious diseases and cancer models^15^. Nevertheless, hu-mice have not been widely used in the study of human autoimmune diseases, and previous attempts to model human SLE in humanised mice met with various limitations^17–19^. Despite its artificial nature of induction, pristane-induced SLE model strongly recapitulates many of the human SLE symptoms in mice. Here we show that pristane injection into hu-mice could induce SLE pathogenesis that is mediated by human immune system, and recapitulate major clinical and immunological features found in SLE patients.

Different hu-mice models could be generated from different sources of human HSCs engrafted to various immunodeficient mice strains. To date, NSG mice used in this study are among the best immunodeficient recipient strains for human cell engraftment: they have a long life span; completely devoid of mature lymphocytes and NK cells; and support stable development of human HSCs into mature and functional immune cells^20^. Engraftment of human CD34^+^ HSCs into irradiated NSG pups, as described here, consistently yielded good human immune cells reconstitution, with an average of 40% human immune cells reconstitution in the blood. The lymphoid tissues were, as expected, largely devoid of mouse immune cells, and were mostly comprised of human immune cells (Supplementary Fig. S1A). Still, residual mouse myeloid cells remain present in NSG, and pristane injection notably induced increased number of mouse immune cells in the spleen and peritoneal lavage (Supplementary Fig. S2). As such, we cannot completely rule out the contribution of the residual mouse myeloid cells in the induction and pathogenesis of SLE. Nevertheless, the absence of mouse mature lymphocytes, together with the lack of kidney damage and mortality when pristane was injected to NSG mice alone, strongly suggest that the residual mouse immune cells did not significantly contribute the SLE pathogenesis and that human immune cells were the key component in the pathogenesis of SLE in hu-mice (Figs 1A, 2A). Still, many approaches to completely eradicate these residual mouse cells are currently being generated, which would tremendously improve the application of hu-mice particularly in the studies of autoimmunity^16^.

Production of anti-nuclear autoantibodies is one of the characteristic features of SLE. Previous attempt to model human SLE by engraftment of PBMCs from SLE patients into immunodeficient mice did not induce increased production of human IgG and anti-dsDNA antibodies^17^. Pristane injection into hu-mice significantly increased the total level of human IgG and IgM, and importantly, induced the production of a wide range of human anti-nuclear autoantibodies, including anti-dsDNA, anti-histone, anti-RNP70, anti-SM and anti-SSA antibodies (Fig. 1B). In the current study, we could not assess the difference in disease severity between hu-mice derived from male and female HSC donors, which would require a much larger cohort of HSC donors. However, there was no significant difference in the level of anti-dsDNA production in male and female pristane-injected hu-mice (Supplementary Fig. S2C, which suggests that gender predominance in SLE is largely attributed to the genotype of the immune system. Furthermore, anti-dsDNA antibodies, one of the definitive SLE diagnostic criteria, could be significantly detected as early as 4 weeks post-pristane injection in hu-mice (Supplementary Fig. S2B). This could be highly beneficial for pre-clinical evaluation of novel therapies against SLE.

Lymphopenia is one of the clinical criteria in SLE diagnosis^2^, and it is detected in more than 90% of patients with active SLE^36^. Consistently, pristane injection into hu-mice induced a dramatic loss of human T cells, B cells and NK cells in the peripheral blood within 12 weeks post-pristane injection (Fig. 3). The exact cause of lymphopenia in SLE patients is still unknown, but it has been attributed to the excess level of type I IFN in these individuals^37^.

Immunological analysis of peripheral blood and lymphoid tissues in pristane injected hu-mice revealed hyperactivation of both B cells and T cells, resembling the systemic chronic inflammation state in SLE patients. Relative expansion of CD27^+^ memory B cells and CD27^−^IgD^−^ population is consistent with observation in patients where B cell lymphopenia in SLE prejudicially affects CD19^+^CD27^−^IgD^+^ naïve B cells (Fig. 4C and Supplementary Fig. S4C)^38^. Additionally, consistent with findings in patients with active SLE^38,39^, increased number and frequency of antibody producing plasmablasts/plasma cells could be found in the peripheral blood and lymphoid tissues of pristane-injected hu-mice (Fig. 4B and Supplementary Fig. S4B). T cell abnormalities in SLE include abnormal signaling transduction, leading to lower threshold of T cells activation^28^ and amplification of inflammation response resulting in organ damage^29^. In concordance to the aberrant T cells activation in SLE, pristane injection into hu-mice induced the activation of both CD4^+^ T cells and CD8^+^ T cells, as shown by accumulation of CCR7^−^CD45RA^−^ effector memory cells, and increased expression of HLA-DR, a human-specific lymphocyte activation marker (Fig. 5). Human CD45^+^ leukocytes were also found infiltrating into the glomeruli, thus contributing to the pathological damage seen in the kidney and other organs (Supplementary Fig. S2D)^40^.

Type I IFN has been shown to play a crucial role in SLE pathogenesis^41^. We were not able to detect increased level of human type I IFN (specifically, IFN-α), nor increased expression of ISGs in the blood of pristane-injected humice (data not shown). This might be attributed to the imperfect development and function of the myeloid subsets in humanised mice^42^. However, we did observe increased expression of human IFN signature genes in human hepatocytes in pristane-injected humice (Fig. 6B). A number of other human pro-inflammatory cytokines were also increased upon pristane injection (Fig. 6A). Increased level of IL-6 is in line with B cell hyperactivation, maturation into plasma cells and induction of autoantibodies production^43^. Production of IFN-γ and IL-17 signify the role of Th1 and Th17 cells in SLE^44^. IL-18 and MCP-1 have both been found to be elevated in the serum/plasma and urine of SLE patients, and was positively correlated to disease activity and renal involvement; both cytokines have been proposed as biomarkers for disease activity and renal involvement^45,46^. IL-8, which has no murine homologue, has been found to be significantly higher in the serum and bronchoalveolar lavage of SLE patients, and its level is positively correlated to disease activity^47,48^. Hu-mice SLE model could be particularly useful for studies of human-specific genes/factors, such as IL-8, in the induction and pathogenesis of SLE and as potential immunotherapy targets. Furthermore, recent advancements in genome editing have opened up huge applications potential with genetic manipulation. Genetic manipulation of human HSCs^49^ to be engrafted into humanised mice could be a revolutionary tool in studying the contribution of a single gene/factor in the pathogenesis of various human diseases, including SLE.

While hu-mice have proven to be instrumental in translational research involving human specific immune responses, several limitations remain in the current generation of humanised mice. As mentioned above, these limitations include the presence of residual mouse myeloid cells; suboptimal myeloid subsets reconstitution and function; and impaired B cells maturation and antibody production. Injection of pristane to hu-mice induced production of anti-nuclear autoantibodies (Fig. 1B), however, the level of these autoantibodies was much lower compared to those in SLE patients. Efforts to improve hu-mice have focused on improving the myeloid subset and adaptive immunity in the humanised immune system, which would particularly enhance the level of antibodies production. Different methods to supplement hu-mice with human-tendentious cytokines such as transgenic/knock-in mice and hydrodynamic injection based gene delivery have been developed to further improve human immune cell functions^42,50^. Future improvements on the adaptive and humoral immune response in hu-mice would certainly further improve the application of hu-mice in the study of human autoimmunity and other human diseases.

Our work provides a proof-of-concept study that hu-mice, particularly those derived from HSC transplantation, is able to recapitulate many key features of SLE immunopathology, and thus useful for SLE research. Furthermore, many human specific immunological responses were observed in this model, including HLA-DR expression on T cells, production of human specific genes (IL-8), and lymphopenia. Thus we anticipate that this new SLE model can provide a closest-to-human *in vivo* platform for the study of human specific mechanisms as well as immunotherapy target identification and evaluation.

## Methods

### Isolation of human CD34^+^ HSCs

Human fetal HSC specimens were obtained from aborted fetuses at 16–23 weeks of gestation in accordance with the institutional ethical guidelines of Kadang Kerbau Women’s and Children’s Hospital (KKH). All patients gave written informed consent for the donation of their fetal tissues for research. Isolation of human CD34^+^ HSCs were as described previously^35^. Briefly, human CD34^+^ HSCs were purified by magnetic-based cell sorting using EasySep CD34-positive selection kit (StemCell Technologies) under sterile condition, with purity of 90% and above. All methods involving human tissue samples were performed in accordance to the relevant guidelines and regulations, with approval from SingHealth and National Health Care Group Research Ethics Committees Singapore (CIRB Ref: 2012/064/B).

### Generation of hu-mice and SLE model

NSG mice were purchased from The Jackson Laboratory, and bred in pathogen-free animal facility at Biological Resource Centre (BRC) in Agency for Science, Technology and Research (A*STAR). All animal experimental procedures were approved and done in accordance to Institutional Animal Care and Use Committee (IACUC) guidelines of A*STAR Singapore. NSG pups within 3 days after birth were sublethally irradiated with 1 Gy γ-ray and transplanted with 1 × 10^5^ human CD34^+^ HSCs by intra-hepatic injections. Human immune cells reconstitution level was determined at 12 weeks post-transplantation by flow cytometry of the peripheral blood, and was calculated as percentage of human CD45^+^ cells among total cells expressing CD45 (both mouse and human).

Less than 6% of the total hu-mice population displayed a human reconstitution level lower than 10%, and were excluded from the study (Supplementary Fig. S1A). A total of 18 NSG and 82 hu-mice (engrafted from 5 different HSC donors) were used in this study, consisting of 8 male and 10 female NSG, and 37 male and 45 female hu-mice. Experimental mice were chosen randomly, regardless of sex and reconstitution level (Supplementary Fig. S1B). Pristane (Sigma Aldrich) was injected intra-peritoneally to 12–13 weeks old hu-mice and NSG.

### Human immunoglobulin (Ig) and autoantibodies measurement

Levels of human IgG, IgM, and anti-nuclear (anti-dsDNA, anti-histone, anti-RNP70, anti-SM and anti-SSA) IgGs in the plasma of control and pristane-injected hu-mice were measured by ELISA quantification kits according to manufacturer’s instruction (From Bethyl laboratories, and Alpha Diagnostic Inc, respectively).

### Histopathology and immunohistochemistry

Lungs and kidneys from control and pristane-injected hu-mice and NSG were harvested at eight weeks post-injection, fixed with 10% formalin and embedded in paraffin for processing into 5 µm tissue sections. Rehydrated lung and kidney sections were stained with Hematoxylin & Eosin (H&E) (Thermo Scientific), and evaluated by a pathologist who was blinded of the samples’ identity. Glomerular enlargement was quantified by area measurement of 50 random glomeruli from kidneys of each experimental animal. Images were captured using brightfield slide scanner (Axio Scan Z1) and processed by Zen software (Carl Zeiss). For immunohistochemistry (IHC), kidney sections were subjected to heat-mediated antigen retrieval with sodium citrate (pH6) buffer prior to staining with appropriate antibodies. IHC staining was performed using the SuperPicture 3^rd^ Gen IHC Detection Kit (Life Technologies) according to manufacturer’s instruction. Primary antibodies used in the study include anti-human IgG, anti-human IgM (Bethyl Laboratories), and anti-human CD45 (AbCAM). Anti-mouse, anti-rabbit and anti-goat HRP conjugated secondary antibodies were purchased from Life Technologies.

### Proteinuria

Urine was collected from control and pristane-injected hu-mice in the morning into a clean eppendorf tube, and when necessary stored in −80 °C for long-term storage. Urine was centrifuged at maximum speed to eliminate any sediment, and supernatant was dropped into urine analysis strip (Uristix, Siemen) according to manufacturer’s instruction.

### Multiplex quantification of human cytokines

Blood plasma and peritoneal lavage fluid were collected from control and pristane-injected hu-mice at eight weeks post-injection. Plasma was collected through centrifugation of peripheral blood, while peritoneal fluid was collected by flushing the peritoneal cavity with 3 ml of PBS/2% FCS. Level of various human cytokines in the plasma and peritoneal lavage fluid was measured using multiplex bead-based cytometry array (BioLegend Legendplex) according to manufacturer’s instruction, acquired on LSR II flow cytometer (BD Biosciences) and data was analysed using Legendplex software.

### Flow cytometry

For flow cytometry analysis, peripheral blood, peritoneal lavage cells and lymphoid tissues from control and pristane-injected hu-mice were processed to obtain single cell suspensions. Spleen, mesenteric lymph nodes and liver were digested with collagenase IV (Gibco) with an addition of DNaseI (Sigma Aldrich) to prevent cell clumping, and filtered to obtain single cells suspension. When necessary, cell suspensions were subjected to red blood cell lysis (Gibco). Cells were stained with fluorescent conjugated antibodies to human cell surface markers, as follows: CD3 (clone UCHT1; BioLegend), CD4 (clone SK3; BD Biosciences), CD8 (clone SK1; BioLegend), CD10 (clone HI10a; BD Biosciences), CD19 (clone SJ25C1; BD Biosciences), CD20 (clone 2H7; BioLegend), CD25 (clone BC96; BioLegend), CD27 (clone L128, BD Biosciences), CD38 (clone HB-7, Biolegend), CD45 (HI30; BioLegend, BD Biosciences), CD45RA (clone HI100; BioLegend), CD56 (clone HCD56; BioLegend), CD69 (clone FN50; BD Biosciences), CD86 (clone FUN-1; BD Biosciences), CD197 (clone A20, BD Biosciences), IgD (clone IA6-2; BioLegend), HLA-DR (clone L243; BD Biosciences) and mouse CD45.1 (clone A20; BD Biosciences and Biolegend). CountBright^TM^ (ThermoFisher) Absolute Counting Beads was used for quantification of absolute cell numbers, according to manufacturer’s instruction. Viable cells were distinguished by DAPI staining, and cell doublets were excluded through FSC-H and FSC-A gating. Samples were acquired on LSR II flow cytometer (BD Biosciences) and analysed with FlowJo (Tree Star). Relative expression level was measured in Staining Index, where the formula is [(MFI (Mean Fluorescence Intensity)_(pos)_/MFI_(neg)_*2 s.d_(neg)_]^51^.

### Liver perfusion and enrichment of human EGFR^+^ hepatocyte

Liver perfusion was done as described previously^35^. Human EGFR^+^ cells were selected using PE selection kit (StemCell Technologies) according to manufacturer’s instruction. The enriched cells were then used for RNA extraction. The purity of enriched EGFR^+^ cells was >90% and the yield of live EGFR^+^ cells recovered from 16 weeks old control and pristine-treated mice ranged from ~400,000 to 800,000 cells/mouse.

### Gene expression profiling by quantitative real-time PCR

RNA was extracted from human EGFR^+^ hepatocyte using the RNeasy Mini kit (Qiagen) and cDNA synthesis was performed using RT kit (Qiagen), with normalized amount of total RNA across samples. Quantitative RT-PCR (qPCR) was performed on Applied Biosystems 7500 real time pCR system (Applied Biosystems) using Sso-Eva-green reagent (Bio-rad) and custom-made primers (IDT) specific for human genes. The gene expression was normalised to house keeping gene, human GAPDH. The list of primers are attached (Supplementary Table S2)

### Statistical Analysis

All statistical tests were performed using 2-tailed unpaired nonparametric test (Mann-Whitney). Survival curve comparison was performed with the Log-Rank (Mantel-Cox) test. Plots and statistical values were built and calculated using GraphPad Prism software. Error bars indicated SEM, and *P* < 0.05 was considered as significant.

## Electronic supplementary material

Supplementary Information
