# Supplementary material for: A Novel Human Systemic Lupus Erythematosus Model in Humanised Mice
Source: Sci Rep. 2017 Nov 30;7:16642. doi: 10.1038/s41598-017-16999-7 (PMC5709358; doi:10.1038/s41598-017-16999-7)
Supplement: Supplementary file 1 — Supplementary Information [file 41598_2017_16999_MOESM1_ESM.pdf]

Title: A Novel Human Systemic Lupus Erythematosus Model in Humanised Mice

Merry Gunawan[1], Zhisheng Her[1], Min Liu[1], Sue Yee Tan[1], Xue Ying Chan[1], Wilson Wei Sheng Tan[1], Shubasree Dharmaraaja[1], Yong Fan[2], Chee Bing Ong[3], Eva Loh[4], Kenneth Tou En Chang[4], Thiam Chye Tan[5], Jerry Kok Yen Chan[6, 7], Qingfeng Chen[1, 2, 8*]

[1]Humanized mouse unit, Institute of Molecular and Cell Biology, Agency for Science, Technology and Research (A*STAR), Singapore; [2]Key Laboratory for Major Obstetric Diseases of Guangdong Province, The Third Affiliated Hospital of Guangzhou Medical University, Guangzhou, 510150, China; [3]Advanced Molecular Pathology Laboratory, Institute of Molecular and Cell Biology, Agency for Science, Technology and Research (A*STAR), Singapore; [4]Department of Pathology and Laboratory Medicine, KK Women's and Children's Hospital, Singapore; [5]Department of Obstetrics & Gynaecology, KK Women's and Children's Hospital, Singapore; [6]Department of Reproductive Medicine, KK Women's and Children's Hospital, Singapore; [7]Experimental Fetal Medicine Group, Yong Loo Lin School of Medicine, National University of Singapore; [8]Department of Microbiology and immunology, Yong Loo Lin School of Medicine, National University of Singapore, Singapore.

* Address correspondence to Qingfeng Chen, Institute of Molecular and Cell Biology, Proteos, 61 Biopolis Drive, Singapore 138673; Tel (+65) 65869873; E-mail: qchen@imcb.a-star.edu.sg

Supplementary figures and tables

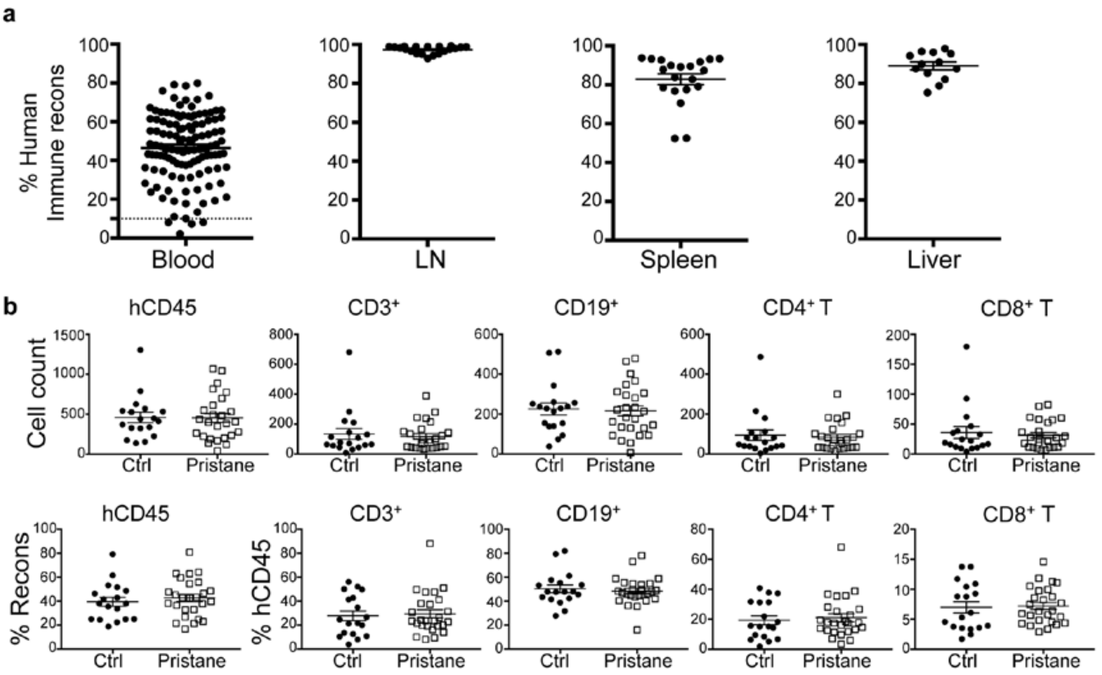

Fig. S1. (**a**) Human immune cells reconstitution level in the peripheral blood and organs of untreated humice from at least three different HSCs were analyzed by flow cytometry. Dotted line on the peripheral blood chart signified humice with human reconstitution level below 10% that was excluded from the study. (**b**) (Top panel) Absolute cell count of human $CD45^{+}$, $CD3^{+}$, $CD19^{+}$, $CD4^{+}$ T cells and $CD8^{+}$ T cells in the peripheral blood of untreated (control) and pristane-treated humice group in the beginning of the experiment (wk0). (Bottom panel) Percentage of human CD45 reconstitution and percentage of human $CD3^{+}$, $CD19^{+}$, $CD4^{+}$ T cells and $CD8^{+}$ T cells from the total human CD45+ cells in the peripheral blood of untreated (control) and pristane-treated humice group in the beginning of the experiment (wk0). Data is from four independent experiments (Ctrl n = 20; pristane n = 26).

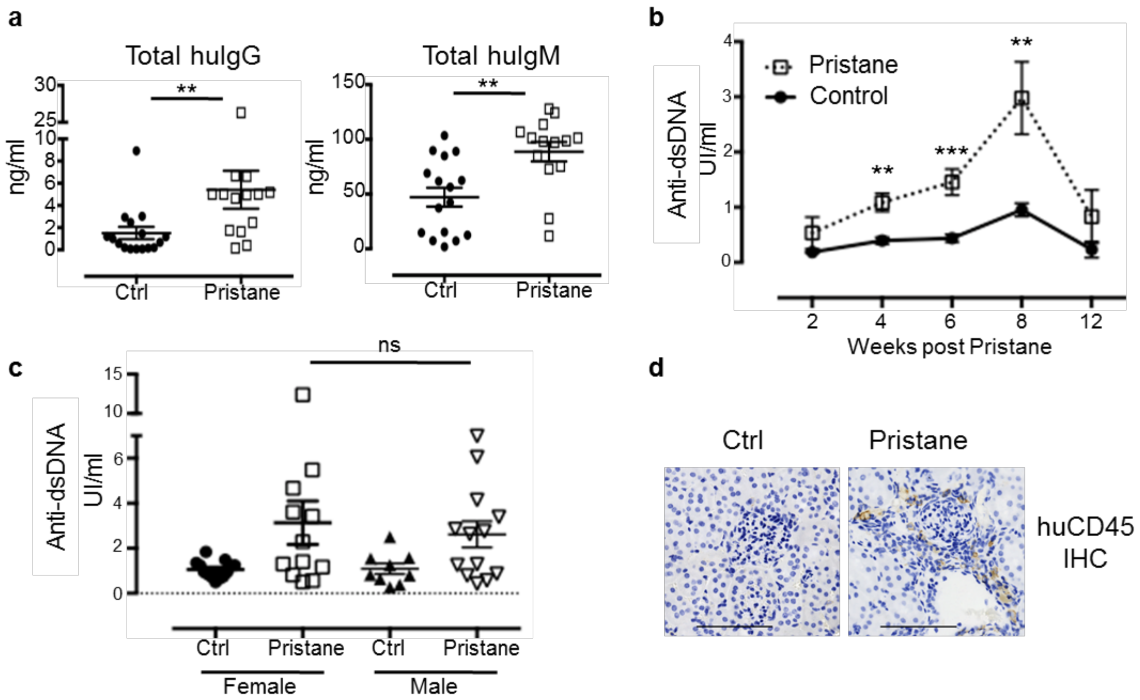

Fig. S2. (**a**) Total human IgG and IgM in the plasma of control and pristane-injected humice at 8 weeks post-injection, as measured by ELISA. (**b**) Level of human anti-dsDNA IgG was measured in control and pristane-injected humice at the indicated time points. (**c**) Level of human anti-dsDNA antibodies in male and female pristane-injected humice at 8 weeks post-injection (**a-c**) Figure shown is from four experiments (control n = 16; pristane n = 14). (**d**) kidney sections of control and pristane-injected humice were IHC stained for human CD45. Image is representative of two independent experiments (control n = 8; pristane n = 10). Scale bar represents 100µm ** *P*<0.001

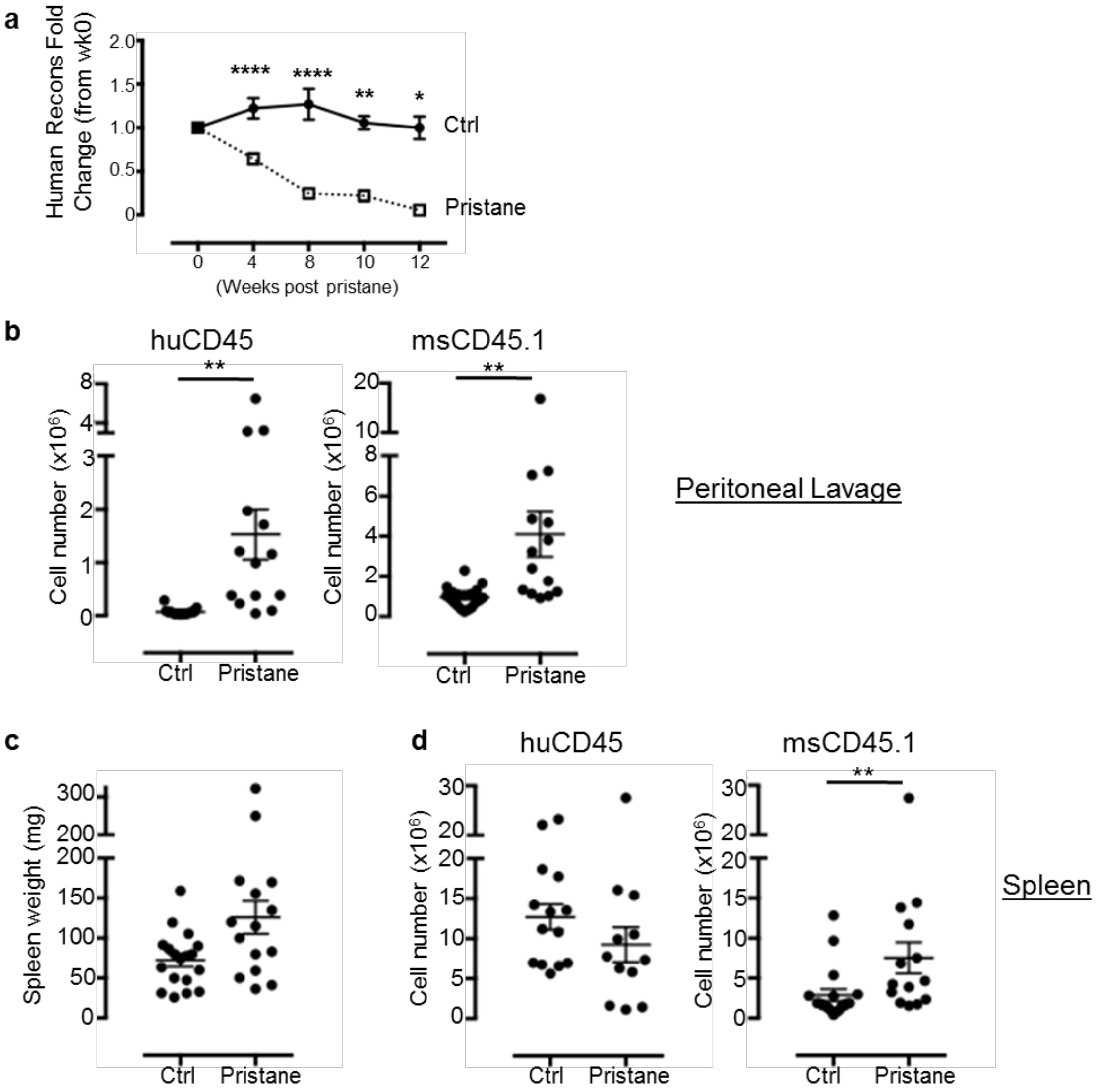

Fig. S3. (a) Human cells reconstitution level in the peripheral blood of control and pristane-injected humice over the indicated time points plotted as fold change to the initial reconstitution level at the beginning of the experiment. (**b**) Absolute cell numbers of $CD45^{+}$ human and $CD45.1^{+}$ mouse immune cells were quantified in the peritoneal lavage of control and pristane-injected humice at 8 weeks post-injection. (**c**) Spleen from control and pristane-injected humice was weighed at 8 weeks post-injection. (**d**) Absolute cell number of $CD45^{+}$ human and $CD45.1^{+}$ mouse immune cells were quantified in the spleen of control and pristane-injected humice at 8 weeks post-injection. Figure shown is from three independent experiments (control n = 14; pristane n = 12). * $P<0.05$; ** $P<0.01$; **** $P<0.0001$.

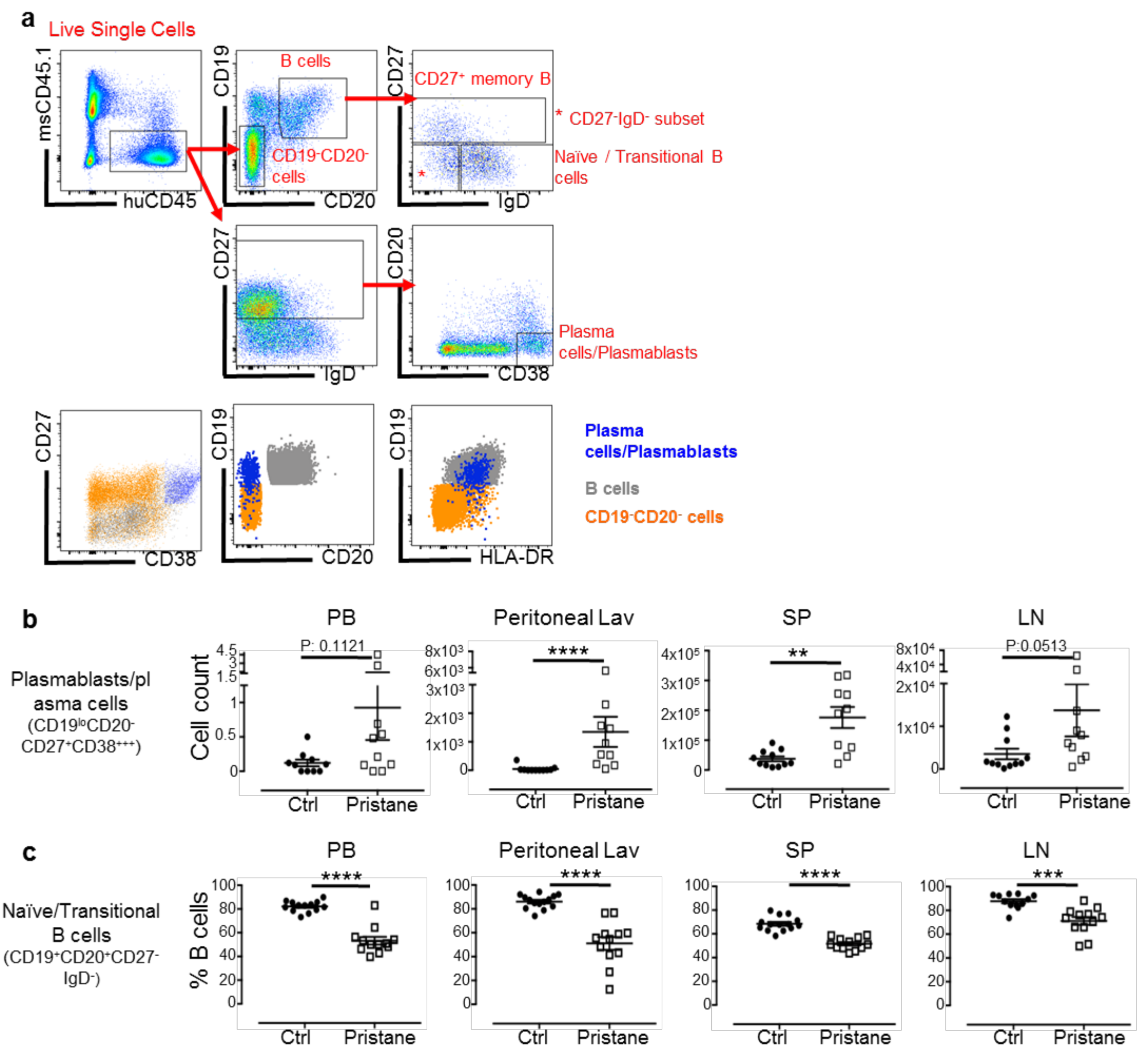

Fig. S4. (**a**) Gating strategy of human B cell subsets: Total B cells, CD27^{+} memory B cells, CD27^{-}IgD^{-} subset, CD27^{-}IgD^{+} naïve/transitional B cells, and CD19^{lo}CD20^{-}CD27^{++}CD38^{+++} plasmablasts/plasma cells. Bottom panel showed surface expression level of the indicated markers on plasma cells/plasmablasts (blue), total B cells (gray) and CD19^{-}CD20^{-} non-B cells population. Figure shown is a representative of the spleen population of pristane-injected humice at week 8 from three independent experiments. (**b**) Absolute cell count of the plasmablast/plasma cells population in control and pristane-injected humice at week 8 post-injection. Figure shown is from three independent experiments (control n = 11; pristane n = 10). (**c**) Proportion of naïve/transitional B cells in control and pristane-injected humice at week 8 post-injection. Figure shown is from three independent experiments (control n = 14; pristane n = 12). * *P*<0.05; ** *P* < 0.01; *** *P*<0.001; **** *P*<0.0001.

		CD4 Naïve		CD4 Central Memory		CD4 Effector Memory		CD4 Effector	
		Mean (SE)	P value	Mean (SE)	P value	Mean (SE)	P value	Mean (SE)	P value
PB	Ctrl	52.46 (5.75)	0.0031 (**)	19.48 (1.88)	0.03542 (n.s)	25.11 (5.36)	0.0031 (**)	2.36 (0.80	0.7242 (n.s)
	Pristane	13.39 (3.73)		22.09 (1.91)		61.60 (3.67)		2.48 (0.89)	
Lav	Ctrl	26.51 (3.24)	0.0007 (***)	27.48 (2.99)	0.0007 (***)	39.15 (4.62)	0.0007 (***)	4.17 (0.58)	0.0007 (***)
	Pristane	1.24 (0.33)		12.11 (1.63)		85.82 (1.93)		0.54 (0.11)	
SP	Ctrl	32.92 (5.60)	0.0047 (**)	15.49 (1.15)	0.2824 (n.s)	44.25 (6.73)	0.0027 (**)	6.47 (1.1)	0.0083 (**)
	Pristane	5.69 (2.7)		12.89 (1.78)		78.48 (3.53)		2.04 (0.73)	
LN	Ctrl	45.56 (3.28)	0.0007 (***)	19.70 (3.18)	0.0426 (*)	29.84 (4.55)	0.0007 (***)	4.74 (1.1)	0.0263 (*)
	Pristane	10.59 (1.81)		28.56 (2.82)		59.01 (1.96)		1.71 (0.32)	

		CD8 Naïve		CD8 Central Memory		CD8 Effector Memory		CD8 Effector	
		Mean (SE)	P value	Mean (SE)	P value	Mean (SE)	P value	Mean (SE)	P value
PB	Ctrl	64.52 (10.78)	0.0016 (**)	8.92 (1.22)	0.2844 (n.s)	23.69 (10.51)	0.0031 (**)	2.86 (0.44)	0.2222 (n.s)
	Pristane	10.51 (2.56)		13.00 (1.96)		74.51 (3.84)		1.97 (0.43)	
Lav	Ctrl	32.11 (7.28)	0.0007 (***)	10.13 (2.08)	0.1079 (n.s)	50.25 (9.13)	0.0007 (***)	7.51 (2.74)	0.0426 (*)
	Pristane	0.84 (0.27)		5.91 (0.91)		92.56 (0.99)		0.70 (0.39)	
SP	Ctrl	56.17 (8.34)	0.0007 (***)	7.76 (0.59)	0.0593 (n.s)	28.73 (7.97)	0.0007 (***)	7.33 (0.67)	0.0007 (***)
	Pristane	5.31 (1.97)		11.54 (1.66)		81.71 (3.22)		1.44 (0.35)	
LN	Ctrl	71.00 (4.90)	0.0007 (***)	7.04 (1.16)	0.0047 (**)	9.16 (2.49)	0.0007 (***)	12.80 (3.99)	0.3450 (n.s)
	Pristane	26.16 (5.74)		14.97 (1.51)		50.64 (5.96)		8.23 (1.61)	

Table S1. Statistical analysis of $CD4^+$ T cell (Top) and $CD8^+$ T cell (bottom) subsets (Fig 5A). Table represents the Mean and S.E.M of naïve, effector, central memory and effector memory T cells in the peripheral blood (PB), peritoneal lavage (Lav), spleen (SP) and lymph node (LN) of control and pristane-injected humice at 8 weeks post-injection. Table is derived from four independent experiments (control n = 18; pristane n = 14). Statistical analysis was performed with Mann-Whitney test.

Gene	Forward primer	Reverse primer
Human GAPDH	GCT TAA CTC TGG TAA AGT GGA TAT	ATG GAA TTT GCC ATG GGT GGA AT
Human MX1	GTT TCC GAA GTG GAC ATC GCA	CTG CAC AGG TTG TTC TCA GC
Human MX2	CAG AGG CAG CGG AAT CGT AA	TGA AGC TCT AGC TCG GTG TTC
Human ISG15	CGC AGA TCA CCC AGA AGA TCG	TTC GTC GCA TTT GTC CAC CA
Human GS3686	AGC CGT CAG GGA TGT ACT ATA AC	AGG GAA TCA TTT GGC TCT GTA GA

Table S2. Primer sequences for relative genes
